# Suppression by γ-Hydroxybutyric Acid of “Alcohol Deprivation Effect” in Rats: Preclinical Evidence of its *anti*-Relapse Properties

**DOI:** 10.3389/fpsyt.2012.00095

**Published:** 2012-11-05

**Authors:** Giancarlo Colombo, Mauro A. M. Carai, Gian Luigi Gessa

**Affiliations:** ^1^Section of Cagliari, Neuroscience Institute, National Research Council of ItalyMonserrato, Italy

**Keywords:** γ-hydroxybutyric acid, alcohol deprivation effect, relapse-like drinking, GABA_B_ receptor, Sardinian alcohol-preferring rats

## Abstract

γ-Hydroxybutyric acid (GHB) reduces (a) alcohol intake and alcohol motivational properties in alcohol-preferring rats and (b) alcohol drinking and craving for alcohol in human alcoholics. The present study was designed to extend to relapse-like drinking the capacity of GHB to suppress different alcohol-related behaviors in alcohol-preferring rats. The “alcohol deprivation effect,” defined as the temporary increase in alcohol intake occurring in laboratory animals after a period of alcohol deprivation, was used as model of alcohol relapse. Acute administration of non-sedative doses of GHB (0, 100, 200, and 300 mg/kg, i.p.) resulted in the complete suppression of the extra-amount of alcohol consumed by Sardinian alcohol-preferring rats during the first hour of re-access to alcohol after a 14-day period of deprivation. These data demonstrate that GHB suppressed relapse-like drinking in a rat model of excessive alcohol consumption.

## Introduction

The short-chain fatty acid, γ-hydroxybutyric acid (GHB; see Snead and Gibson, 2005; Agabio et al., 2010), has been used for years – in some European Countries – in the treatment of alcohol dependence. Several open and double-blind clinical surveys indicate indeed that GHB administration reduced alcohol craving and consumption, promoted abstinence, and ameliorated alcohol withdrawal syndrome in alcoholics. Specifically, a small double-blind study (Gallimberti et al., 1992) and some subsequent open studies (Addolorato et al., 1996, 1998; Moncini et al., 2000; Maremmani et al., 2001; Caputo et al., 2003, 2007, 2011) indicated that treatment with GHB was effective in reducing alcohol drinking, promoting abstinence, and controlling craving for alcohol (for review, see Agabio and Gessa, 2002; Addolorato et al., 2009). Further, three double-blind studies (Gallimberti et al., 1989; Addolorato et al., 1999a; Nimmerrichter et al., 2002) and some additional open studies (Nava et al., 2007; Elsing et al., 2009) reported that treatment with GHB was also effective in suppressing the symptomatology of alcohol withdrawal syndrome (for review, see Agabio and Gessa, 2002; Addolorato et al., 2009). In terms of mechanism of action, the similarity of the pharmacological profile of GHB and alcohol has led to hypothesize that the suppressing effects exerted by GHB on alcohol withdrawal syndrome, alcohol consumption, and craving for alcohol may be due to the “substitution” of alcohol actions, similar to methadone in heroin addiction (see Agabio and Gessa, 2002; Addolorato et al., 2009). As predictable on the basis of a number of clinical and preclinical observations (see Nicholson and Balster, 2001; Drasbek et al., 2006), the major limitation of the therapeutic use of GHB appears to be its abuse potential: a portion of patients undergoing GHB treatment voluntarily increased their daily GHB dosage (although some of these self-administered increases were made in an attempt to achieve more effective therapeutic doses) (Addolorato et al., 1996; Gallimberti et al., 2000; Glisson and Norton, 2002; Caputo et al., 2011) and some patients even developed dependence on GHB (Addolorato et al., 1999b; see also Caputo et al., 2009).

Treatment with GHB reduced several alcohol-related behaviors also in laboratory rodents. Specifically, administration of non-sedative doses of GHB markedly reduced voluntary alcohol intake in selectively bred, Indiana alcohol-preferring (June et al., 1995) and Sardinian alcohol-preferring (sP) (Agabio et al., 1998) rats exposed to the standard, homecage two-bottle “alcohol vs. water” choice regimen. Additionally, administration of non-sedative doses of GHB reduced the motivational properties of alcohol (the animal correlate of human craving for alcohol), measured in sP rats exposed to sessions of operant, oral alcohol self-administration under the progressive ratio schedule of reinforcement (Maccioni et al., 2008). Treatment with non-sedative doses of GHB also reduced alcohol-seeking behavior, measured in sP rats initially trained to lever-respond for alcohol under a standard procedure of oral alcohol self-administration and then exposed, in the test session, to extinction responding (non-reinforced lever-responding was used as index of alcohol-seeking behavior) (Maccioni et al., 2008). Finally, administration of GHB resulted in the complete suppression of the severity of alcohol withdrawal signs in rats made physically dependent on alcohol by the repeated, forced administration of intoxicating doses of alcohol (Fadda et al., 1989).

The present study was designed to extend to alcohol relapse-like drinking the investigation on the capacity of GHB to suppress different alcohol-related behaviors in rats. To this end, sP rats were exposed to the “alcohol deprivation effect” (ADE) paradigm, a validated animal model of relapse episodes occurring in human alcoholics. Relapse to heavy alcohol drinking, together with loss of control over alcohol, represent the core features of alcohol addiction in humans (see Morse and Flavin, 1992; Kleber et al., 2007). ADE is defined as the temporary increase in alcohol drinking occurring after a period of alcohol abstinence (see Spanagel, 2005; Martin-Fardon and Weiss, 2013). Rats of the sP line constitute a good experimental model for investigations on ADE. Indeed, sP rats display robust, although transient, increases in alcohol intake after a period of alcohol deprivation of at least 1 week: over the first hour of re-access to alcohol, alcohol intake in sP rats is usually double than that recorded in control, alcohol-non-deprived rats (e.g., Agabio et al., 2000; Serra et al., 2003).

## Materials and Methods

All experimental procedures employed in the present study were in accordance with the Italian Law on the “Protection of animals used for experimental and other scientific reasons.”

### Animals

Male sP rats (see Colombo et al., 2006a; Bell et al., 2012), from the 71st generation and 75-days-old at the start of the study, were used. Rats were individually housed in standard plastic cages with wood chip bedding. The animal facility was under an inverted 12:12 h light-dark cycle (lights on at 23:00), at a constant temperature of 22 ± 2°C, and relative humidity of approximately 60%. Rats were extensively habituated to handling and intraperitoneal injections. Standard rat chow (Mucedola, Settimo Milanese, Italy) was always available.

### Experimental procedure

Rats (*n* = 64) were continuously offered alcohol (10% v/v, in water) and water under the standard, homecage two-bottle choice regimen, with unlimited access for 24 h/day and for four consecutive weeks. Alcohol, water, and food intake was monitored once a day by weighing the bottles and food pellets (0.1-g accuracy) immediately before the onset of the dark phase. Bottles were refilled every day with fresh solution and their left-right positions interchanged daily.

At the end of the 4-week period of access to alcohol and water, during which rats consumed daily an average of approximately 6 g/kg alcohol, rats were divided into two groups (*n* = 32) matched for body weight as well as alcohol and water intake over the last 7 days. One rat group was deprived of alcohol for 14 consecutive days, during which water was the sole fluid available (alcohol-deprived rats). The second rat group continued to have unlimited access to alcohol and water (alcohol-non-deprived rats), with the exception of the last 6 h before GHB injection, when the alcohol bottle was removed to ensure that blood alcohol levels were equal to zero at the time of the test.

At the end of the 14th day of the deprivation phase, rats of both groups were divided into four subgroups (*n* = 8), matched for body weight, and injected acutely and intraperitoneally with 0, 100, 200, and 300 mg/kg GHB. GHB (sodium salt; Laboratorio Farmaceutico CT, Sanremo, Italy) was dissolved in distilled water [3.4% w/v; this concentration (kept fixed in the three doses of GHB) was chosen to minimize tissue irritation at the injection site (e.g., Maccioni et al., 2008)] and administered 10–15 min before lights off. Control rats (0 mg/kg GHB) were treated with an equal volume of saline. Alcohol was represented at lights off. Alcohol, water, and food intake was recorded 60 min later (i.e., the time interval during which sP rats display the most pronounced ADE; e.g., Agabio et al., 2000; Serra et al., 2003).

### Data analysis

Data on the effect of treatment with GHB on alcohol, water, and food intake during the 60-min observation period were expressed in g/kg, ml/kg, and g/kg, respectively, and analyzed by separate two-way (deprivation; treatment) ANOVAs, followed by the Newman–Keuls test for *post hoc* comparisons.

## Results

**Figure 1:**
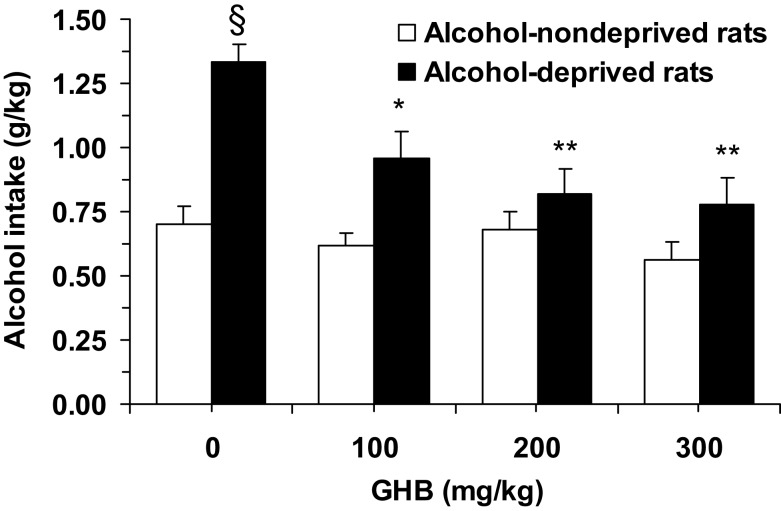
**Suppressing effect of the acute administration of γ-hydroxybutyric acid (GHB) on “alcohol deprivation effect” in Sardinian alcohol-preferring (sP) rats**. Alcohol-deprived rats were initially allowed to consume alcohol (10% v/v, in water) and water under the homecage two-bottle choice regimen with unlimited access for 24 h/day and four consecutive weeks, and then deprived of alcohol for 14 consecutive days; conversely, alcohol-non-deprived rats had continuous access to alcohol and water (with exception of the last 6 h before GHB injection, when the alcohol bottle was removed to ensure that blood alcohol levels were equal to zero at the time of the test). Food pellets were always available. About 10–15 min before representation of the alcohol bottle (which coincided with lights off), rats from both groups were injected with GHB (0, 100, 200, and 300 mg/kg; i.p.). Alcohol intake was registered 60 min after lights off. Each bar is the mean ± SEM of *n* = 8. ^S^*P* < 0.0005 with respect to alcohol-non-deprived rats receiving 0 mg/kg GHB (Newman–Keuls test); *P < 0.005 and **P < 0.0005 with respect to alcohol-deprived rats receiving 0 mg/kg GHB (Newman–Keuls test).

ANOVA indicated significant effects of both deprivation [*F*(1, 56) = 31.99, *P* < 0.0001] and treatment with GHB [*F*(3, 56) = 6.44, *P* < 0.001], and a significant interaction [*F*(3, 56) = 3.48, *P* < 0.05], on alcohol intake during the first hour of the post-deprivation phase. Alcohol intake in vehicle-treated alcohol-deprived rats was approximately 90% higher than that recorded in vehicle-treated alcohol-non-deprived rats (Figure 1), indicative of the development of ADE. *Post hoc* analysis indicated that all doses of GHB suppressed ADE, as alcohol intake in all GHB-treated alcohol-deprived rat groups was significantly lower than that recorded in vehicle-treated alcohol-deprived rats (Figure 1); further, when alcohol-deprived and -non-deprived rats treated with each GHB dose were compared, no significant difference was recorded (Figure 1).

Finally, neither water [*F*_deprivation_(1, 56) = 0.78, *P* > 0.05; *F*_treatment_(3, 56) = 0.57, *P* > 0.05] nor food [*F*_deprivation_(1, 56) = 0.05, *P* > 0.05; *F*_treatment_(3, 56) = 1.02, *P* > 0.05] intake resulted to be altered by GHB treatment (Table 1).

**Table 1:** **Lack of effect of the acute administration of γ-hydroxybutyric acid (GHB) on water and food intake in Sardinian alcohol-preferring (sP) rats exposed to the “alcohol deprivation effect” paradigm**.

GHB (mg/kg)	Water intake (ml/kg)		Food intake (g/kg)	
	Alcohol-non-deprived rats	Alcohol-deprived rats	Alcohol-non-deprived rats	Alcohol-deprived rats
0	0.96 ± 0.19	1.35 ± 0.14	6.86 ± 0.67	7.03 ± 0.80
100	0.89 ± 0.13	1.50 ± 0.43	4.83 ± 0.86	7.06 ± 0.37
200	1.38 ± 0.36	1.49 ± 0.47	6.13 ± 0.91	5.90 ± 0.66
300	1.64 ± 0.34	1.35 ± 0.15	8.39 ± 0.69	5.72 ± 1.18

*Each value is the mean ± SEM of *n* = 8*.

*Alcohol-deprived rats were initially allowed to consume alcohol (10% v/v, in water) and water under the homecage two-bottle choice regimen with unlimited access for 24 h/day and four consecutive weeks, and then deprived of alcohol for 14 consecutive days; conversely, alcohol-non-deprived rats had continuous access to alcohol and water (with exception of the last 6 h before GHB injection, when the alcohol bottle was removed to ensure that blood alcohol levels were equal to zero at the time of the test). Food pellets were always available. About 10–15 min before representation of the alcohol bottle (which coincided with lights off), rats of both groups were injected with GHB (0, 100, 200, and 300 mg/kg; i.p.). Water and food intake was registered 60 min after lights off*.

## Discussion

The results of the present study indicate that acute treatment with GHB suppressed the extra-amount of alcohol (ADE) voluntarily consumed by alcohol-preferring sP rats after a 2-week period of forced abstinence from alcohol. As ADE has been proposed and validated as an animal model of relapse episodes occurring in human alcoholics (see Spanagel, 2005; Martin-Fardon and Weiss, 2013), these data extend to relapse-like drinking the capacity of GHB to suppress different alcohol-related behaviors, including alcohol drinking under the two-bottle choice regimen (June et al., 1995; Agabio et al., 1998), operant oral alcohol self-administration (Maccioni et al., 2008), and alcohol-seeking behavior (Maccioni et al., 2008), in alcohol-preferring rats. These data are also in close agreement with the results of a number of clinical surveys reporting the capacity of GHB to promote and maintain alcohol abstinence and to prevent alcohol relapse (Gallimberti et al., 1992; Addolorato et al., 1996, 1998; Moncini et al., 2000; Maremmani et al., 2001; Caputo et al., 2003, 2007).

In the present study, treatment with GHB did not alter, even minimally, food intake in either alcohol-deprived or -non-deprived rats. These data allow to reasonably exclude that suppression of ADE was secondary to any sedative effect of GHB, that would have resulted in a non-specific reduction of all consummatory behaviors. Water intake was also unaltered by treatment with GHB; an increase in water intake (theoretically compensating GHB-induced reduction in alcohol intake) was not expected, as total fluid intake (the sum of alcohol solution and water consumed) in vehicle-treated alcohol-deprived rats was largely higher than that required to satisfy the rats’ fluid needs and treatment with GHB suppressed only the extra-amount of alcohol solution consumed because of the previous alcohol deprivation.

Data from alcohol-non-deprived rats indicate a lack of effect of treatment with GHB on regular alcohol drinking over the 60-min recording period. These results are consonant with those of a previous study that temporally characterized the effect of GHB on alcohol drinking in sP rats (Agabio et al., 1998): in the latter study, acute administration of GHB (given at doses identical to those used in the present study) to sP rats continuously exposed to the two-bottle “alcohol vs. water” choice regimen dose-dependently reduced alcohol intake only at the 15- and 30-min recording times (i.e., taking into account the pretreatment time, 25–30 and 40–45 min after GHB administration); at the 60-min recording time (i.e., the same time interval of the present study), the reducing effect of GHB on alcohol intake had already completely vanished (Agabio et al., 1998).

The GABA_B_ receptor constitutes the major, although not the sole, site of action of GHB. Brain concentrations of GHB – easily reachable after administration of behaviorally active doses of GHB (as those used in the present study) – bind directly to the GABA_B_ receptor, exerting agonistic properties (Mathivet et al., 1997; Lingenhoehl et al., 1999; for review, see Bernasconi et al., 2002). Accordingly, the results of the present study may be interpreted suggesting that GHB suppressed relapse-like drinking *via* activation of the GABA_B_ receptor. This interpretation is in line with the results of recent studies demonstrating that the GABA_B_ receptor agonists, baclofen and CGP 44532, dose-dependently suppressed ADE in sP rats (Colombo et al., 2003, 2006b; Carai et al., 2005).

## Conflict of Interest Statement

The authors declare that the research was conducted in the absence of any commercial or financial relationships that could be construed as a potential conflict of interest.
